# Regulation of Stemness in Carcinoma Cells

**DOI:** 10.1155/2017/7681769

**Published:** 2017-07-27

**Authors:** Jijun Hao, Juli Unternaehrer, Xiaojiang Cui, Ninghui Cheng, Yusuke Oji

**Affiliations:** ^1^College of Veterinary Medicine, Graduate College of Biomedical Sciences, Western University of Health Sciences, Pomona, CA 91766, USA; ^2^Division of Biochemistry, School of Medicine, Loma Linda University, Loma Linda, CA 92350, USA; ^3^Department of Surgery, Samuel Oschin Cancer Institute, Cedars-Sinai Medical Center, Los Angeles, CA 90048, USA; ^4^Department of Pediatrics, Baylor College of Medicine, Houston, TX 77030, USA; ^5^Department of Functional Diagnostic Science, Osaka University Graduate School of Medicine, Osaka 5650871, Japan

Over the last decade, the important roles of cancer stem cells (CSCs) in tumor recurrence and metastasis have been increasingly recognized. Significant efforts have been made to understand the mechanisms underlying regulation of CSCs' stemness and biology with the goal of developing effective therapies to target CSCs for cancer treatment.

The purpose of this special issue is to provide readers with a representative outlook of the recent advances in the CSC research field. The topics cover signaling pathways, transcription factors/epigenetics, miRNA, and microenvironment in the regulation of CSC stemness as well as identification of CSC markers.

This special issue publishes seven selected papers regarding the above specific timely topics, and the details are summarized below.

Signaling regulation and epigenetic modification are believed to play key roles in CSCs' stemness and functions. J. Koury et al. focused on three critical evolutionarily conserved signaling pathways (Wnt, Hedgehog, and Notch pathways) and their crosstalk in governing CSCs' fate and summarized therapeutic studies targeting these pathways to eliminate CSCs and improve overall cancer treatment outcomes. In addition, G. M. Kelly and M. I. Gatie reviewed current available knowledge of transcription factors, DNA methylation, and chromatin remodeling in embryonal carcinoma cells (ECCs). Furthermore, the roles of miRNA in ECCs and rhabdomyosarcoma (RMS) have been discussed, respectively, in G. M. Kelly and M. I. Gatie's and A. J. Hron and A. Asakura's articles.

Compelling evidence indicates that tumor microenvironment is a key regulator in maintenance of CSC stemness, invasiveness, and drug resistance. P. M. Aponte and A. Caicedo reviewed the organization of tumor microenvironment components with a focus on mesenchymal stem/stromal cells (MSCs), followed by therapeutic strategies targeting CSCs in tumors. In parallel, E. Y.-T. Lau et al. offered a thorough overview of stromal cells, immune cells, extracellular matrix, tumor stiffness, and hypoxia in the regulation of CSC plasticity and therapeutic resistance.

CSCs normally constitute a very small proportion of total tumor cells. Thus, identifying reliable and specific CSC markers will help develop effective therapies to precisely target and destroy the CSCs. In prostate cancer, numerous markers are postulated to be associated with prostate CSCs; however, the clinical significance of these markers remains largely unproven. In the article titled “Prostate Cancer Stem Cell Markers Drive Progression, Therapeutic Resistance, and Bone Metastasis”, K. S. Harris and B. A. Kerr reviewed current prostate CSC markers with functional relevance linked to cancer progression, metastatic colonization and growth, recurrence, or therapeutic resistance. Moreover, X. Song et al. reported that the cells with CD19, CD45, and CD44 surface markers identified in atrial myxoma were CSC-like cells and may have the capacity for myxoma initiation and progression.

The guest editors hope this special issue provides readers with helpful information of recent advances in CSC research and may stimulate interest in further research in this area.

